# Galectin-3 and fibulin-1 in systolic heart failure - relation to glucose metabolism and left ventricular contractile reserve

**DOI:** 10.1186/s12872-016-0437-6

**Published:** 2017-01-10

**Authors:** Pernille Holmager, Michael Egstrup, Ida Gustafsson, Morten Schou, Jordi S. Dahl, Lars Melholt Rasmussen, Jacob E. Møller, Christian Tuxen, Jens Faber, Caroline Kistorp

**Affiliations:** 1 Department of Medicine, Endocrine Unit, Herlev University Hospital, Herlev, Denmark; 2 Faculty of Health and Medical Sciences, Copenhagen University, Copenhagen, Denmark; 3 Department of Cardiology, Rigshospitalet, Copenhagen, Denmark; 4 Department of Cardiology, Hvidovre University Hospital, Hvidovre, Denmark; 5 Department of Cardiology, Herlev University Hospital, Herlev, Denmark; 6 Department of Cardiology and Cardiothoracic Surgery, Odense University Hospital, Odense, Denmark; 7 Department of Clinical Biochemistry and Pharmacology, Odense University Hospital, Odense, Denmark; 8 Centre of Individualized Medicine in Arterial Diseases, Odense University Hospital, Odense, Denmark; 9 Department of Cardiology, Odense University Hospital, Odense, Denmark; 10 Department of Cardiology, Bispebjerg University Hospital, Copenhagen, Denmark; 11 Department of Endocrinology, Herlev Hospital, Herlev, Denmark

**Keywords:** Diabetes, Myocardial fibrosis, Galectin-3, Fibulin-1, Left ventricular contractile reserve

## Abstract

**Background:**

Heart failure (HF) patients with diabetes (DM) have an adverse prognosis and reduced functional capacity, which could be associated with cardiac fibrosis, increased chamber stiffness and reduced left ventricular (LV) contractile reserve. Galectin-3 (Gal-3) and fibulin-1 are circulating biomarkers potentially reflecting cardiac fibrosis. We hypothesize that plasma levels of Gal-3 and fibulin-1 are elevated in HF patients with DM and are associated with reduced LV contractile reserve in these patients.

**Methods:**

A total of 155 patients with HF with reduced ejection fraction underwent a low-dose dobutamine echocardiography and blood sampling for biomarker measurements. Patients were classified according to history of DM and an oral glucose tolerance test (OGTT) as: normal glucose tolerance (NGT) (*n* = 70), impaired glucose tolerance (IGT) (*n* = 25) and DM (*n* = 60).

**Results:**

Galectin-3 levels were elevated in DM patients as compared to non-diabetic patients (*P* = 0.02), while higher fibulin-1 levels were observed in HF patients with IGF and DM (*P* = 0.07). Reduced LV contractile reserve was associated with increasing Gal-3 levels (β = −0.19, *P* = 0.03) although, this association was attenuated after adjustment for estimated glomerular filtration rate (*P* = 0.66). Fibulin-1 was not associated with LV contractile reserve (*P* = 0.71).

**Conclusions:**

Galectin-3 and fibulin-1 levels were elevated in HF patients with impaired glucose metabolism. However, reduced LV contractile reserve among HF patients with DM does not to have an independent impact on plasma Gal-3 and fibulin-1 levels.

## Background

Patients with heart failure with reduced ejection fraction (HFrEF) and concomitant diabetes (DM) have a considerably increased mortality rate and reduced exercise capacity compared to non-diabetic HFrEF patients [1]. It is well-established that in HFrEF DM is frequent, and screening with an oral glucose tolerance test (OGTT) reveals a prevalence of more than 30% [2]. We have previously reported that the co-existence of DM, whether it is by history or newly diagnosed is associated with a reduced left ventricular (LV) contractile reserve assessed by low-dose dobutamine echocardiography (LDDE) among HFrEF patients [3]. Further, reduced LV contractile reserve is predictive of prognosis and thus could contribute to the unfavorable outcome of diabetic HFrEF patients [4]. The pathophysiological processes leading to reduced LV contractile reserve remain to be fully elucidated but have been associated with myocardial fibrosis in patients with cardiomyopathy [5].

Galectin-3 (Gal-3) is a galactoside-binding protein which is released to the circulation by activated macrophages and is considered a biomarker of cardiac fibrosis and remodeling [6]. In the myocardium, Gal-3 is primarily expressed in the fibroblasts and in macrophages and plays an important role in the formation of myocardial fibrosis through activation of fibroblasts [6]. In DM increased plasma Gal-3 levels have been associated with both macro- and microvascular complications [7]. Moreover, high levels of Gal-3 are reported in patients with chronic HFrEF and renal dysfunction [8]. Hence, we hypothesize that elevated Gal-3 is associated with reduced LV contractile reserve found among HFrEF patients with DM.

The novel promising biomarker fibulin-1 is emerging as a potential biomarker of cardiomyopathy [9]. Fibulin-1 is an extracellular matrix glycoprotein and is considered to reflect pathological accumulation of extracellular matrix components surrounding vascular smooth muscle cells [10]. To our knowledge no previous studies of fibulin-1 in HFrEF patients have been performed. In patients with chronic renal dysfunction high levels of fibulin-1 are associated with increasing age, HbA1c and creatinine [11]. Plasma fibulin-1 levels have been investigated in patients with aortic stenosis, where an association with decreased longitudinal systolic LV function has been observed [12]. The effects of HFrEF and abnormal glucose metabolism on circulating fibulin-1 levels have, to our knowledge, not been examined previously.

Therefore, the aims of this study were to evaluate the impact of DM and abnormal glucose metabolism on plasma Gal-3 and fibulin-1 concentrations, and further to investigate whether Gal-3 and fibulin-1 levels were associated with LV contractile reserve in HFrEF patients according to presence of DM.

## Methods

### Study population

Between May 2008 and June 2010 155 patients were recruited from the HF clinic at Frederiksberg University Hospital, Copenhagen, Denmark. The cohort has previously been described in detail [3]. Patients were enrolled in the present study if LV ejection fraction (LVEF) was ≤ 45% by echocardiography and baseline measurements of Gal-3 and fibulin-1 were available.

Patients were classified as having DM either by history or DM newly diagnosed by an oral glucose tolerance test (OGTT). The OGTT was performed in patients without known DM within 2 weeks after the baseline visit by ingestion of 75 g glucose dissolved in 250 mL water. Plasma glucose was measured in the fasting state (fasting plasma glucose, FPG) and after 2 h (2hPG). Glycemic status was defined according to WHO diagnostic criteria: Normal glucose tolerance (NGT): FPG <6.1 mmol/L and 2hPG <7.8 mmol/L. Impaired glucose tolerance (IGT): FPG <7.0 mmol/L and 2hPG between 7.8 and 11.1 mmol/L. DM: FPG >7.0 mmol/L and/or 2hPG >11.1 mmol/L.

### Echocardiography

Resting echocardiography and LDDE (as described in detail previously [3]) was performed within 2 weeks after referral to the outpatient clinic on a standard ultrasound machine (IE-33, Philips Inc.) and stored for later analysis [3]. The Doppler analyses were based on an average of five beats for patients with sinus rhythm and 10 beats for patients with atrial fibrillation (AF). LVEF was calculated by the biplane method from apical four- and two-chamber views [13].

### Laboratory analysis

After a minimum 8-h overnight fast and 20 min of supine rest, venous blood was obtained for measurement. Blood was drawn into EDTA tubes, promptly centrifuged at 4 °C, and plasma was frozen at −80 °C in aliquots until laboratory analyses were performed. Insulin resistance was estimated using the Homeostasis Model of Assessment-Insulin Resistance (HOMA-IR): Fasting Glucose (mmol/L) x Insulin (mU/L)/22.5. Galectin-3 was determined using an ELISA kit (BG Medicine, Waltham, USA) [14]. The lower detection limit was 1.32 ng/mL, intra- and interassay coefficient of variation (CV) <6% and <10%, respectively [14]. The fibulin-1 double-antibody sandwich ELISA has previously been described extensively [15]. Antibodies are a mouse anti-fibulin monoclonal 5D12/H7 IgG and goat anti-rabbit IgG alkaline phosphatase [16]. The assay has a lower detection limit of 1.56 μg/mL, an intra- and interassay CV <8% and ~10%, respectively [15].

### Statistical analysis

Distribution of the biomarkers Gal-3 and fibulin-1 in the total study population is depicted in histograms. Continuous variables are presented as mean (SD) or as median (interquartile range), as appropriate. The groups were compared using independent sample *T*-test or Kruskall-Wallis’ test if the assumption of Gaussian distribution was not met. Fibulin-1 levels were normally distributed, while GAL-3 levels were not. The IGT group was small, and we previously demonstrated that LV contractile reserve is reduced in patients with known or new DM but not affected by IGT. Hence, we divided the population in two groups: DM (new or known), and non-DM (NGT or IGT) in the multivariable linear regression analysis. The associations between Gal-3, fibulin-1 and HbA1c were depicted in scatter plots. Multivariable linear regression analyses examining the association between Gal-3 and fibulin-1 levels with LV function were performed. The multivariable regression analyses included co-variables considered of having potential impact of LV contractile reserve in univariable analysis: Age, sex, presence of ischemic heart disease (IHD), estimated glomerular filtration rate (eGFR), atrial fibrillation (AF) and DM status [3]. Further, the relationship between Gal-3, fibulin-1 and parameters of glucose metabolism as HbA1c and DM were examined in multivariable models including confounders associated with Gal-3 and fibulin-1 at the *P* <0.1 level in univariable analysis. Multivariable linear regression analysis was performed as stepwise analyses with backward elimination. Logarithmic transformation of Gal-3 and fibulin-1 were used in the linear regression analyses to meet the assumptions of linearity. Statistical analyses were performed using the SPSS 22 package.

## Results

**Fig. 1:**
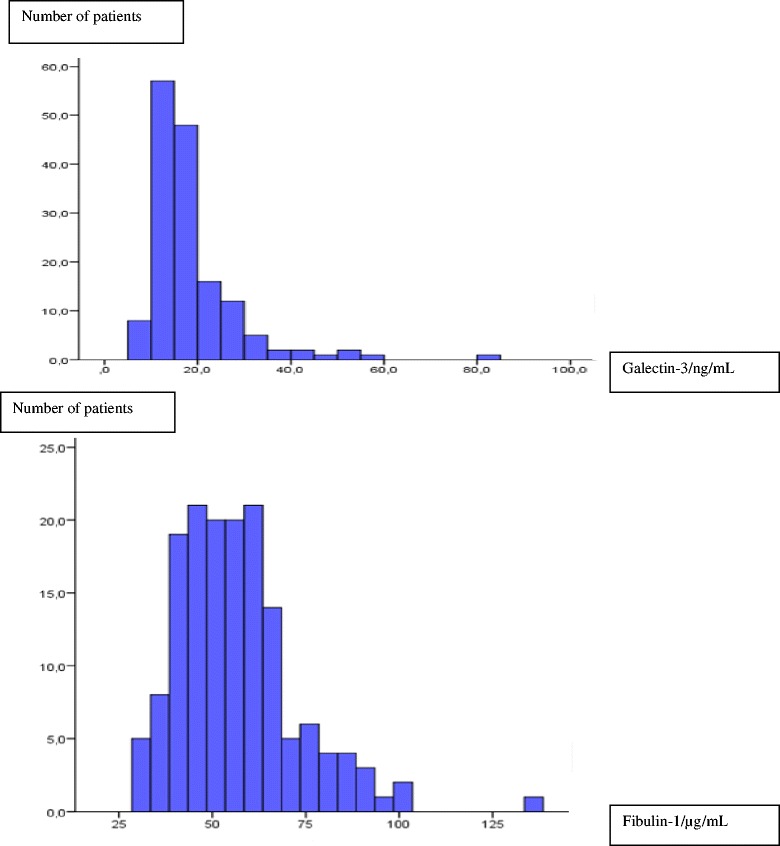
Histograms of Galectin-3 and Fibulin-1

According to the OGTT and history of DM, 70 (45%) of the HF patients had NGT, 25 (16%) had IGT, and 60 (39%) DM, of these 28 (18%) were newly diagnosed. Baseline characteristics according to DM status are presented in Table 1. There were no significant differences with regard to age, gender distribution, eGFR, and IHD between HF patients with and without DM. LVEF at rest (*P* = 0.003), LV contractile reserve (*P* = 0.01), and systolic longitudinal tissue velocity (S’) during LDDE (*P* = 0.03) were reduced among patients with DM compared to the non-diabetic patients. Distributions of Gal-3 and of fibulin-1 are displayed in Fig. 1. Median (IQR) Gal-3 was 16.0 (12.8–21.0) ng/mL, and mean fibulin-1 was 57 ± 16 μg/mL. The univariable associations between the biomarkers and clinical parameters are shown in Table 2. Galectin-3 levels were strongly associated with impaired renal function (eGFR) (β = −0.49, *P* < 0.001) and (β = −0.46, *P* < 0.001) after full adjustment. Fibulin-1 levels were correlated to eGFR, NT-proBNP, presence of AF, and age (Table 2) and remained correlated to eGFR (β = −0.26, *P* < 0.001) after full adjustment.

**Table 1:** Baseline characteristics according to diabetes status

Demographics	DM (*n* = 60)	Non-DM (*n* = 95)	*P* value
Age, years	70.8 (9.6)	69.7 (10.8)	0.54
Sex (female/male), %	32/68	33/67	0.90
IHD, n (%)	40 (67)	52 (55)	0.20
NYHA I + II vs. III + IV, %	77/23	85/15	0.21
AF, n (%)	18 (30)	21 (22)	0.30
Systolic BP, mmHg	134 (110–151)	135 (120–146)	0.58
Heart rate, beats/min	73 (66–81)	69 (60–81)	0.03
*Metabolism*			
BMI, kg/m^2^	28.1 (5.4)	26.3 (5.0)	0.05
FPG, mmol/L	6.9 (1.4)	5.7 (0.5)	<0.001
2hPG (mmol/l)	11.7 (2.9)	6.6 (1.9)	<0.001
HbA1C, mmol/mol	49 (12)	40 (4)	<0.001
HOMA-IR	2.8 (1.5–5.8)	1.7 (1.0–2,8)	<0.001
Total cholesterol, mmol/L	4.0 (1.0)	4.4 (1.1)	0.02
Estimated GFR, ml/min/1.73 m^2^	68 (26)	74 (19)	0.10
*Biomarkers*			
NT-proBNP, pg/mL	1111 (418–2227)	771 (310–1854)	0.08
Gal-3, ng/ml	17.9 (12.9–26.1)	15.6 (12.6–19.3)	0.02
Fibulin-1, μg/ml	60 (19)	55 (13)	0.07
*Echocardiographic variables*			
LVEF rest, %	34 (29–41)	39 (33–45)	0.003
S’ (rest), cm/s	5.2 (4.5–6.5)	5.7 (4.8–6.4)	0.10
E, cm/s	81.6 (62.2–99.7)	72.9 (60.0–92.4)	0.31
e’, cm/s	5.9 (4.6–7.5)	6.6 (5.5–8.7)	0.03
E/e’ (rest) per unit	12.2 (9.6–16.5)	10.2 (8.1–15.4)	0.007
LVEF stress, %	40 (34–47)	49 (40–57)	<0.001
LV contractile reserve, %	7 (2–10)	10 (4–15)	0.01
S’ (stress), cm/s	8.3 (6.3–9.6)	7.3 (5.7–8.5)	0.03
*Medical treatment*			
ACE/AngII-inhibitors, n, (%)	85 (89)	48 (80)	0.10
β-blocker, n (%)	53 (88)	72 (76)	0.05
Loop diuretic dose, mg/24 h	60 (0–80)	0 (0–60)	0.001

Oral glucose tolerance test was only performed in the non-diabetic HF patient. 2hPG: 2 h plasma glucose. *AF* Atrial fibrillation; *BMI* Body mass index; *BSA* Body surface area *E* Peak early diastolic transmitral flow velocity; *e’*; early diastolic longitudinal tissue velocities; *FPG* Fasting plasma glucose; *Gal-3* Galectin-3; *GFR* Glomerular filtration rate; *HOMA-IR* Homeostatic model assessment of Insulin Resistance. *IHD* Ischemic heart disease; *LV* left ventricular; *LVEF* Left ventricular ejection fraction; *NT-proBNP* N-terminal pro B-natriuretic peptide; *NYHA* NYHA functional class; *S’* Systolic longitudinal tissue velocity

**Table 2:** Associations of galectin-3 and fibulin-1 levels with clinical and glucometabolic parameters

	Galectin-3		Fibulin-1	
Parameter	β (SE)	*P*-Value	β (SE)	*P*-Value
Age, per year	0.16 (0.23)	0.05	0.36 (0.12)	<0.001
Sex	0.04 (0.07)	0.59	−0.09 (2.75)	0.29
Ischemic heart disease	−0.01 (0.07)	0.90	−0.009 (2.71)	0.92
NYHA I + II vs. III + IV	0.16 (0.09)	0.06	0.08 (3.37)	0.34
Atrial fibrillation	0.17 (0.07)	0.03	0.17 (2.83)	0.03
Diabetes mellitus	0.27 (0.07)	0.001	0.16 (2.62)	0.05
eGFR, per ml/min/1.73 m^2^	−0.49 (0.10)	<0.001	−0.33 (0.06)	<0.001
*Metabolism*				
BMI, per kg/m^2^	−0.04 (0.006)	0.63	−0.12 (0.25)	0.14
FPG, per mmol/L	0.19 (0.02)	0.02	−0.07 (1.37)	0.44
2hPG, per mmol/L	0.15 (0.01)	0.09	0.26 (0.42)	0.004
HbA1C, per mmol/mol	0.25 (0.16)	0.02	0.21 (0.14)	0.009
HOMA-IR, per unit	0.17 (0.01)	0.06	−0.08 (0.52)	0.36
*Biomarkers*				
NT-proBNP per pg/mL	0.30 (0.12)	<0.001	0.38 (2.24)	<0.001

Univariable regression models

*2hPG* 2 h plasma glucose, *BMI* Body mass index, *FPG* Fasting plasma glucose, *GFR* Glomerular filtration rate, *HOMA-IR* Homeostatic model assessment of insulin resistance, *NT-proBNP* N-terminal pro B-natriuretic peptide, *NYHA* NYHA functional class

### Galectin-3, fibulin-1 and relationship with glucose metabolism

**Fig. 2:**
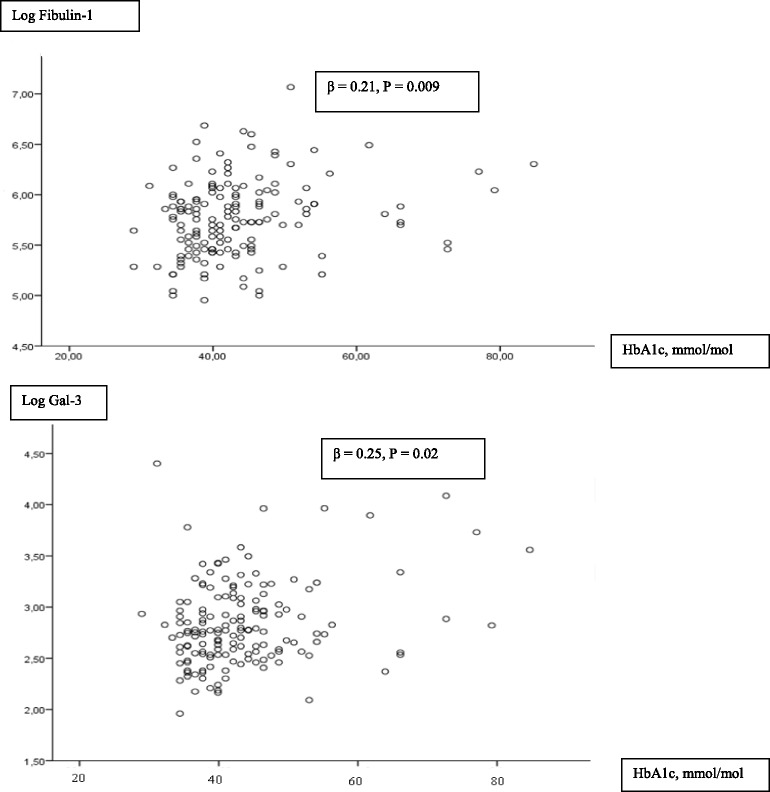
Scatter plot of HbA1c, Galectin-3 and fibulin-1 levels

Plasma Gal-3 levels were elevated among HF patients with DM (17.9 (12.9 26.1) ng/mL) and IGT (16.8 (14.1–22.1) ng/mL) as compared with NGT patients 15.2 (11.6–18.3) ng/mL (*P* = 0.02) (Table 1). No difference was found in patients classified as IGT compared to NGT (*P* = 0.38). The relationship between the presence of DM and Gal-3 levels was further investigated in a multivariable model adjusting for age, eGFR, AF, and NYHA functional class, being confounders associated with Gal-3 at the *P* < 0.1 level. In this model DM was independently associated with elevated Gal-3 (β = 0.17, *P* = 0.02). An impact of glucose metabolism on Gal-3 levels was further observed by a positive association with HbA1c (β = 0.25, *P* = 0.02) (Fig. 2), and FPG (*P* = 0.02), but only borderline significantly associated with 2hPG and HOMA (Table 2). HbA1c was entered in a multivariable model, and demonstrated to have an independent relation with increasing Gal-3 levels (β = 0.16, *P* = 0.02). After full adjustment, no association between Gal-3 and FPG (*P* = 0.56, data not shown) was observed.

There was only a trend towards increased plasma fibulin-1 levels when comparing in HF patients with DM vs non DM (*P* = 0.07), although impaired glucose metabolism assessed by presence of IGT and DM were associated with equivalent elevated mean fibulin-1 levels 61 ± 16 μg/mL vs. 60 ± 19 μg/mL, respectively compared to HF patients with NGT 52 ± 13 μg/mL (*P* = 0.01). Further, HbA1c was associated with increasing fibulin-1 concentrations (β = 0.21, *P* = 0.009) (Fig. 2), and this relation was not markedly attenuated after adjustment for the confounders age, AF, and eGFR (β = 0.15, *P* = 0.05). Fibulin-1 levels which were more than 2SD from mean Finulin-1 levels were considered outliers. Hence, we excluded 3 measurements of Fibulin-1 levels which were considered outliers. The exclusion of the outliers did not change the results (data not shown).

### Galectin-3, fibulin-1 and relationship with LV function

The univariable associations between plasma Gal-3 and fibulin-1 levels with echocardiographic parameters obtained at rest and during LDDE are displayed in Table 3. There was an association between Gal-3 and LV contractile reserve (β = −0.19, *P* = 0.03) and Gal-3 and LVEF during stress (β = −0.25, *P* = 0.003). In a multivariable regression model the association with LV contractile reserve was no longer significant (*P* = 0.66), as only DM (β = −0.20, *P* = 0.03) and eGFR (β = −0.18, *P* = 0.04) remained predictive of LV contractile reserve after complete adjustment (data not shown).

**Table 3:** Associations of galectin-3 and fibulin-1 levels with echocardiographic parameters

	Galectin-3		Fibulin-1	
	β (SE)	*P*-Value	β (SE)	*P*-Value
*Resting echocardiography*				
LVEF, per %	−0.15 (0.14)	0.08	−0.24 (0.14)	0.004
S’, per cm/s	−0.15 (0.14)	0.07	−0.25 (0.02)	0.002
*Stress echocardiography*				
LVEF stress, per %	−0.25 (0.14)	0.003	−0.20 (0.003)	0.02
LV contractile reserve, per %	−0.19 (0.06)	0.03	−0.03 (0.005)	0.71
S’ (stress), per cm/s	−0.15 (0.14)	0.07	−0.24 (0.01)	0.004

Univariable regression analyses

*LV* left ventricular/left ventricle, *LVEF* Left ventricular ejection fraction, *S’* Systolic longitudinal tissue velocity

Finally, the patients were divided into two groups; normal and low eGFR (<60 mL/min/1.73 m^2^). In both groups, no association between Gal-3 and LV contractile reserve (β = −0.06, *P* = 0.57 in patients with normal eGFR vs. β = −0.02, *P* = 0.95 in patients with low eGFR) was demonstrated. With regard to fibulin-1, resting LVEF, LVEF and S’ during stress were all associated with increasing levels of this biomarker in a univariable analysis, as presented in Table 3. Yet, after adjustment for relevant co-variables including eGFR no independent associations between fibulin-1 and these LV systolic measures were found. An independent relation of fibulin-1 with decreasing eGFR (β = −0.24, *P* = 0.004) and with age (β = 0.22, *P* = 0.008) was observed. Again, the patients were divided into two groups; normal and low eGFR (<60 mL/min/1.73 m^2^). In both groups, no association between fibulin-1 and LV contractile reserve (β = 0.06, *P* = 0.55 in patients with normal eGFR vs. β = 0.02, *P* = 0.89 in patients with low eGFR) was presented. We have performed analyses where the patients with known diabetes were separated from the patients with newly diagnosed diabetes. This, however, did not influence the results.

## Discussion

The primary findings of the present study were that plasma Gal-3 and fibulin-1 levels were elevated in HFrEF patients with abnormal glucose metabolism compared to HFrEF patients with NGT. The impact of impaired glucose metabolism on both Gal-3 and fibulin-1 levels was underlined by an independent association with increasing HbA1c. However, increased Gal-3 and fibulin-1 levels were not associated with the reduced contractile reserve observed among diabetic HFrEF patients.

Median Gal-3 level was 16.0 ng/mL, which is similar to findings in comparable HFrEF populations and clearly increased compared to healthy subjects [17, 18]. The mean fibulin-1 level in our HF population was 57 ± 16 μg/mL, which is slightly higher than the mean level observed in a DM population (46 ± 1.3 μg/mL [19]). Fibulin-1 is a novel biomarker and high concentrations have primarily been reported in patients with aortic stenosis and DM compared to a level of 5.6 (4.1–8.4) μg/mL reported in healthy men [11, 20, 21].

The current results suggest that glucose metabolism is associated with circulating Gal-3 concentrations in HF, as elevated levels were found in patients with DM and an association with increasing HbA1c was demonstrated. The observations were supported by two previous studies reporting increased concentrations in patients with type 2 DM [18, 21]. However, we found a significant impact of renal function on plasma Gal-3 levels suggesting that other parameters, such as DM complications like diabetic nephropathy were more predictive of Gal-3 levels in HFrEF patients.

With regard to fibulin-1 we found elevated concentrations among patients with IGT and DM compared to NGT and a positive association with HbA1c, which corresponds with previous findings among type 2 DM reporting elevated circulating levels and of an up-regulation of fibulin-1 in the arterial walls [19, 22].

To our knowledge, the present study is the first to evaluate the relation between circulating Gal-3 and fibulin-1 levels and cardiac function evaluated by LDDE. Galectin-3 was not associated with LV contractile reserve and LVEF during low dose dobutamine infusion after adjusting for relevant confounders, especially eGFR. The present study demonstrated that impaired renal function was a strong and independent predictor of elevated Gal-3 levels, which is in line with previous studies in patients with DM and in a community cohort [8, 23]. The elimination of Gal-3 has to our best knowledge not been described, but the present findings indicate a renal component. The gold standard for evaluation of myocardial fibrosis requires an endomyocardial biopsy, which is a high-risk procedure. Cardiac magnetic resonance imaging (CMR) is another method that seems to provide a good estimate of myocardial fibrosis [24, 25]. However, it is technically difficult to perform CMR in patients with AF and devices; which are highly prevalent in HFrEF patients. Furthermore, the association between cardiac fibrosis estimated by CMR and Gal-3 levels is not well described. In patients with previous myocardial infarction Gal-3 was moderately associated with LVEF and infarct size [26]. We report no association of the biomarkers Gal-3 with IHD. A proposed mechanism could be that former MI in our HFrEF patients are not associated with persisting increased activity of the fibrotic tissue and thereby secretion to the circulation of these biomarkers.

Myocardial fibrosis evaluated by histomorphometry and LV contractile reserve seems associated in patients with dilated cardiomyopathy [5]. In patients with acute decompensated HF, the PRIDE study reported a relationship between Gal-3 and resting echocardiographic parameters related to diastolic function [27]. In the current cohort of patients with chronic HFrEF we found no independent association with parameters related to diastolic function (data not shown).

We found no association between fibulin-1 levels and LV contractile reserve, while univariable associations were demonstrated between fibulin-1 and resting parameters LVEF and S’. However, no association was shown in the multivariable analyses. This is partly in accordance with data from patients with aorta stenosis, where fibulin-1 levels were related with S’, but not with LVEF [10]. Furthermore, we demonstrated a moderate, inverse association with eGFR which could blunt a potential weaker association with LV contractile reserve.

### Limitations

This is a cross-sectional study. Hence, conclusion regarding causality is not possible. A larger sample size would have allowed us to investigate the IGT group in more detail.

## Conclusion

Galectin-3, but not fibulin-1 levels are elevated in diabetic compared to non-diabetic HFrEF patients. Both Gal-3 and fibulin-1 levels are independently and positively associated with HbA1c, but not with LV contractile reserve in HF patients.

## Abbreviations

2hPG: 2 h plasma glucose
AF: Atrial fibrillation
BMI: Body mass index
BSA: Body surface area
CMR: Cardiac magnetic resonance imaging
DM: Diabetes mellitus
E: Peak early diastolic transmitral flow velocity
e’: Early diastolic longitudinal tissue velocities
eGFR: Estimated glomerular filtration rate
FPG: Fasting plasma glucose
Gal-3: Galectin 3
GFR: Glomerular filtration rate
HF: Heart failure
HFrEF: Heart failure with reduced ejection fraction
HOMA-IR: Homeostasis Model of Assessment-Insulin Resistance
IGT: Impaired glucose tolerance
IHD: Ischemic heart disease
LDDE: Low dose dobutamine echocardiography
LV: Left ventricular
LVEF: Left ventricular ejection fraction
MI: Myocardial infarction
NGT: Normal Glucose tolerance
NT-proBNP: N-terminal pro B-natriuretic peptide
NYHA: NYHA functional class
OGTT: Oral Glucose Tolerance Test
S’: Systolic longitudinal tissue velocity
SD: Standard deviation
